# High Out-of-Pocket Health Spending in Countries With a Mediterranean Connection

**DOI:** 10.3389/fpubh.2018.00145

**Published:** 2018-05-23

**Authors:** Simon Grima, Jonathan V. Spiteri, Mihajlo Jakovljevic, Carl Camilleri, Sandra C. Buttigieg

**Affiliations:** ^1^Insurance Department, Faculty of Economics Management and Accountancy, University of Malta, Msida, Malta; ^2^University of Kragujevac, Serbia and Lund University, Lund, Sweden; ^3^Economics Department, Faculty of Economics Management and Accountancy, University of Malta, Msida, Malta; ^4^Department of Health Services Management, Faculty of Health Sciences, University of Malta, Msida, Malta; ^5^Health Services Management Centre, School of Social Policy, College of Social Sciences, University of Birmingham, Birmingham, United Kingdom

**Keywords:** European, Mediterranean connection, out-of-pocket health spending, National Health Systems, Healthcare

## Abstract

In this study, we analyzed healthcare provision and health expenditure across six Mediterranean countries that adopt the National Health System (Beveridge model) and that form part of the European Union (EU) with the main aim being that of analyzing and comparing out-of-pocket health spending in countries with a European Mediterranean connection. To this end, we considered various economic indicators and statistics to derive commonalities and differences across these countries and also compared trends in these indicators to those observed across the rest of the EU. We then analyzed these findings in light of other data related to the quality of healthcare delivery and the infrastructure of the health system and discussed recent developments in healthcare within each country and the main challenges faced by the respective health systems. The results show that on average, Mediterranean countries spend less on total healthcare expenditure (THE) than the EU average, both as a proportion of GDP, as well as in per capita terms. This is primarily driven by lower-than-EU-average public funding of healthcare. The 2008/2009 macro-economic and financial crisis had a significant impact on the countries under review, and explains the persistent reductions in public health spending as part of the austerity measures put in force across sectors. On the flipside, Mediterranean countries have a higher presence of private health providers in total funding, thereby explaining the higher Out-of-Pocket (OOPs) health expenditures in these countries relative to the EU-average. With regard to the overall health infrastructure in these countries, we observed that although the supply of physicians is largely in line with the rest of the EU, there is under-supply when it comes to hospital beds. This may be symptomatic of lower government funding. Nonetheless, all countries score highly in the evaluation of the quality of health services, as recorded by international rankings like the WHO's 2000 metric, whereas health system performance indicators, namely mortality rates and life expectancy reveal favorable health outcomes in the Mediterranean EU countries. The findings in this paper may be seen in light of the Mediterranean region's lifestyle in terms of diet, health behavior, health beliefs and shared culture. In particular, the higher out-of-pocket expenditure may reflect the tendency for one-to-one relationships with private clinicians and the pursuit of person-centered care (1). Additionally, the Mediterranean people may not be as disciplined as their European counterparts in accessing and using the public health sector. The lower THE also reflects the fact that the Mediterranean countries are less wealthy than the more economically-advanced European countries.

## Introduction

In most countries, the level of expenditure on healthcare is increasing over time, largely due to their allotment of a higher proportion of the national financial budget (2–4). In fact, various factors have and are contributing to the growth in countries' health care expenditure, which has outpaced inflation over the past decades. This expenditure has more than doubled even though the average world life expectancy has only increased by 3 years over the same period (1990–2008) (5). In the European Union (EU), this trend is most striking since its development is recorded as higher than in other countries. The actual factors contributing to this observation are embedded in the societal changes that have crept in over the years. These include prosperity diseases and aging population (6, 7), which impact the structures of healthcare and the budgets set to cover the health needs of the countries' citizens.

Various theoretical and empirical studies such as those by Ogura and Jakovljevic (6) and Dieleman et al. (3), have examined the linkages among different groups of countries' health spending. The contribution of this paper is to specifically focus on European countries with a Mediterranean connection, especially in relation to out-of-pocket spending, that have a similar health system model of, namely the (Beveridge) National Health System (NHS), which is provided and financed by the government through tax payments (8). Almost all hospitals and clinics are owned by the government. Doctors tend to be employed with the government, although some work entirely privately and others work privately on top of their government job (9). Specifically, the countries in this study are Spain, Cyprus, Greece, Italy, Malta, and Portugal (the latter also included due to its proximity with Spain), which do not fall within any other group studied so far. Purposefully, we have excluded France and Croatia in view of their health system being modeled on the [Bismarck] Social Security Health System. However, we decided to include Portugal in view of its NHS model as well as its proximity to Spain.

Across the Mediterranean countries (including Portugal), health expenditure varies considerably and as in other parts of the world, the demand on healthcare in this region is on the rise. Since the 2008 macro-economic crisis, health spending was markedly reduced across the EU after years of continuous growth. The substantial variation in the distribution of health expenditure across European and Mediterranean countries is reflected in different types of expenditure, whether public, private or insurance-based. The Mediterranean countries adopted a number of measures to contain healthcare costs, namely changes to public funding, the introduction of user charges, changes in entitlements, the introduction of health technology assessment and the restructuring of healthcare services [e.g., 10–12)].

In our comparative research of these countries, we aim to demonstrate the extent to which structure and finances vary or are similar, especially in relation to out-of-pocket spending in the health sector. We explore whether the countries in this region offer similar health services and have similar influences in terms of culture, traditions, climate and diet. In this manner, we will be able to determine whether these influences play a major part in the decisions related to health expenditure. Moreover, the paper aims to uncover trends over time in these countries that may be useful for developed, developing and underdeveloped countries for future readiness and forecasts (13).

## Method

The methodology of this research was based on a detailed analysis and evaluation of a series of data indicators related to health expenditure patterns in each country. Indicators related to health expenditure reflect performance of health systems, albeit the extent to which health expenditure could influence health system efficiency is still debatable (8). We have also utilized formal government releases, policy documents and literature in peer-reviewed journals as required for this research in order to provide context to the data. Quantitative data used in this paper were drawn from the WHO's Global Health Expenditure Database (14), to ensure both reliability and consistency. We used world standards such as total health expenditure (THE) (aggregated, private and public) in US$, the value of Out-of-Pocket (OOPS) health expenditure per person in US$ and Gross Domestic Product (GDP) in US$ Purchase Price Parity ($PPP) per capita. All available data date back to 1995, with the latest reported date being in 2014, enabling a review spanning nearly two decades so as to adequately demonstrate trends. The data set covers countries which, although within the Mediterranean region, stand at different stages of economic evolution and financial system progress, thereby providing a more representative sample owing to their inherent diversity in financial standing. The use of charts helps in providing clarity in the comparison of these countries and across time.

## Results

National expenditure on healthcare has trended upwards for the last quarter of the twentieth century, noting spiked investment in the industry from both the private and public sectors (15). An increase in insurance companies is an indicator of this renewed interest in the healthcare sector (16).

**Figure 1:**
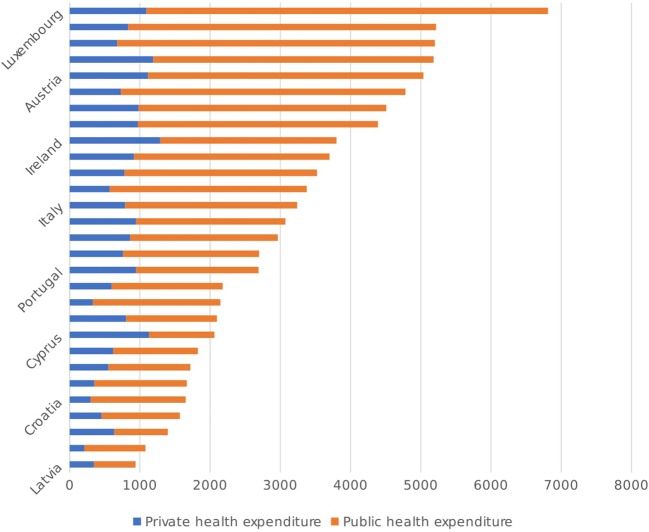
Public and private funding of the healthcare systems in the EU, in PPP$ per Capita [Source: (14)].

**Figure 2:**
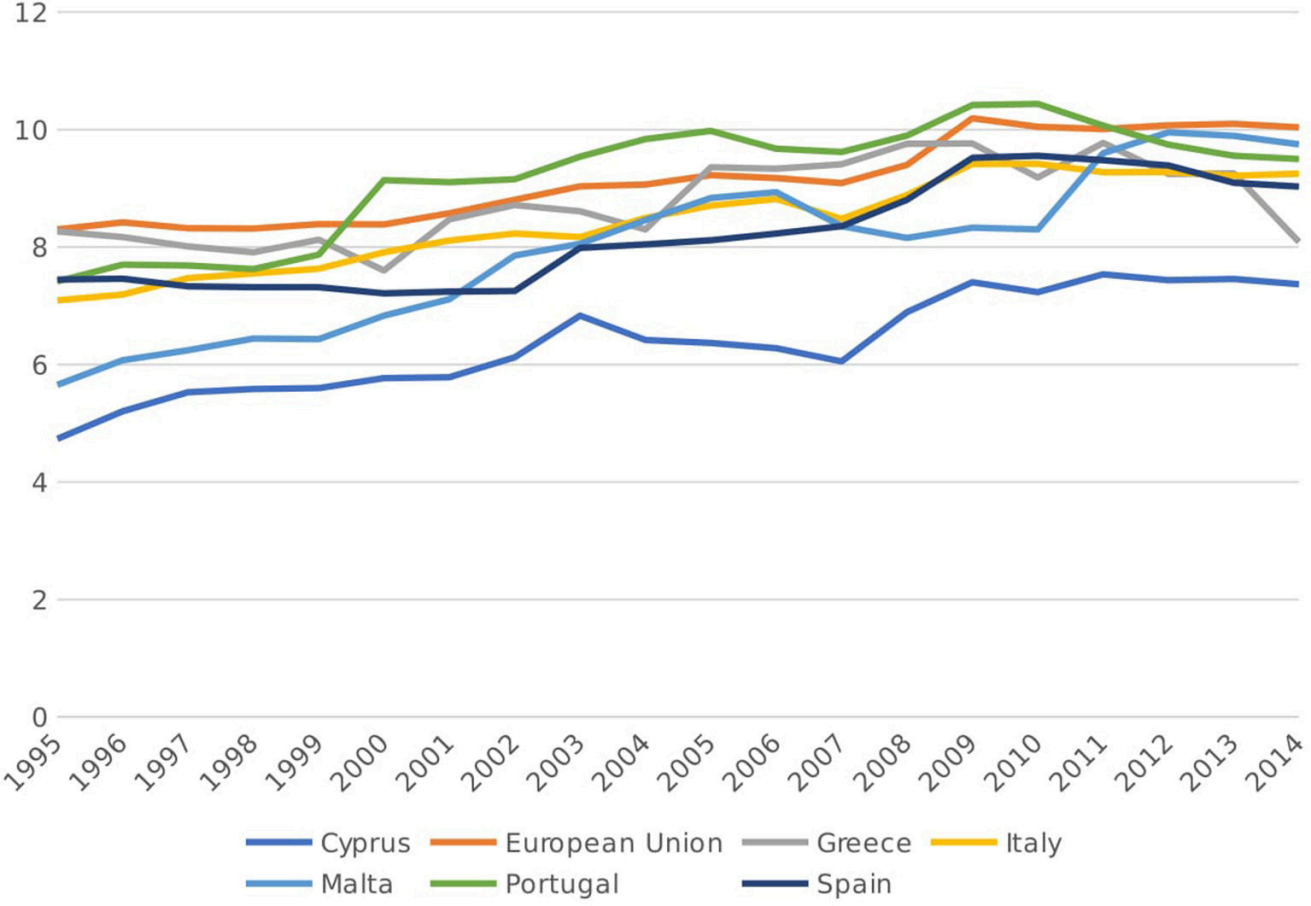
Total health expenditure, as a% of GDP, 1995-2014 [Source: (14)].

Figure 1 shows total per capita health expenditure (in constant 2011 US$, adjusted for PPP) across all European Union countries in 2014, subdivided according to funding source (i.e., private vs. public). As seen from the diagram, all of the Mediterranean countries are below the EU average when it comes to per capita health expenditure, with Italy being the closest and Cyrus being the furthest. Mediterranean countries also spend a lower proportion of their GDP on healthcare relative to the EU average, as seen in Figure 2. Interestingly, all six countries under review report a higher than average level of per capita private health expenditures relative to the rest of the EU, both in absolute terms and as a percentage of THE. Conversely, the Mediterranean countries lag behind when it comes to public health expenditures per capita, which implies that government spending on health services in these countries is lower than the EU average and is in turn being complemented by private care spending. In line with this observation, the Mediterranean countries under consideration also have higher-than-average out-of-pocket (OOP) health expenditures relative to the rest of the EU, as seen in Table 1, with Malta leading the way at $709.02 per capita, some $200 more than the EU average of $502.01. This is particularly important since OOPs may impose a significant financial burden on citizens of a country, given that such payments are not reimbursed by either the government or the private sector (17). Whether any of these factors have had a significant impact on the quality of healthcare provision, or indeed the availability of adequate health-related infrastructure, remains to be seen and requires further analysis.

**Table 1:** 2014 out of pocket health expenditure per Capita, US$ [Source: (14)].

**Country**	**Out of pocket (OOP) health expenditure per capita (US$, 2014)**
Cyprus	$659.83
Greece	$609.57
Italy	$690.11
Malta	$709.02
Portugal	$564.22
Spain	$638.28
European Union	$502.01

**Figure 3:**
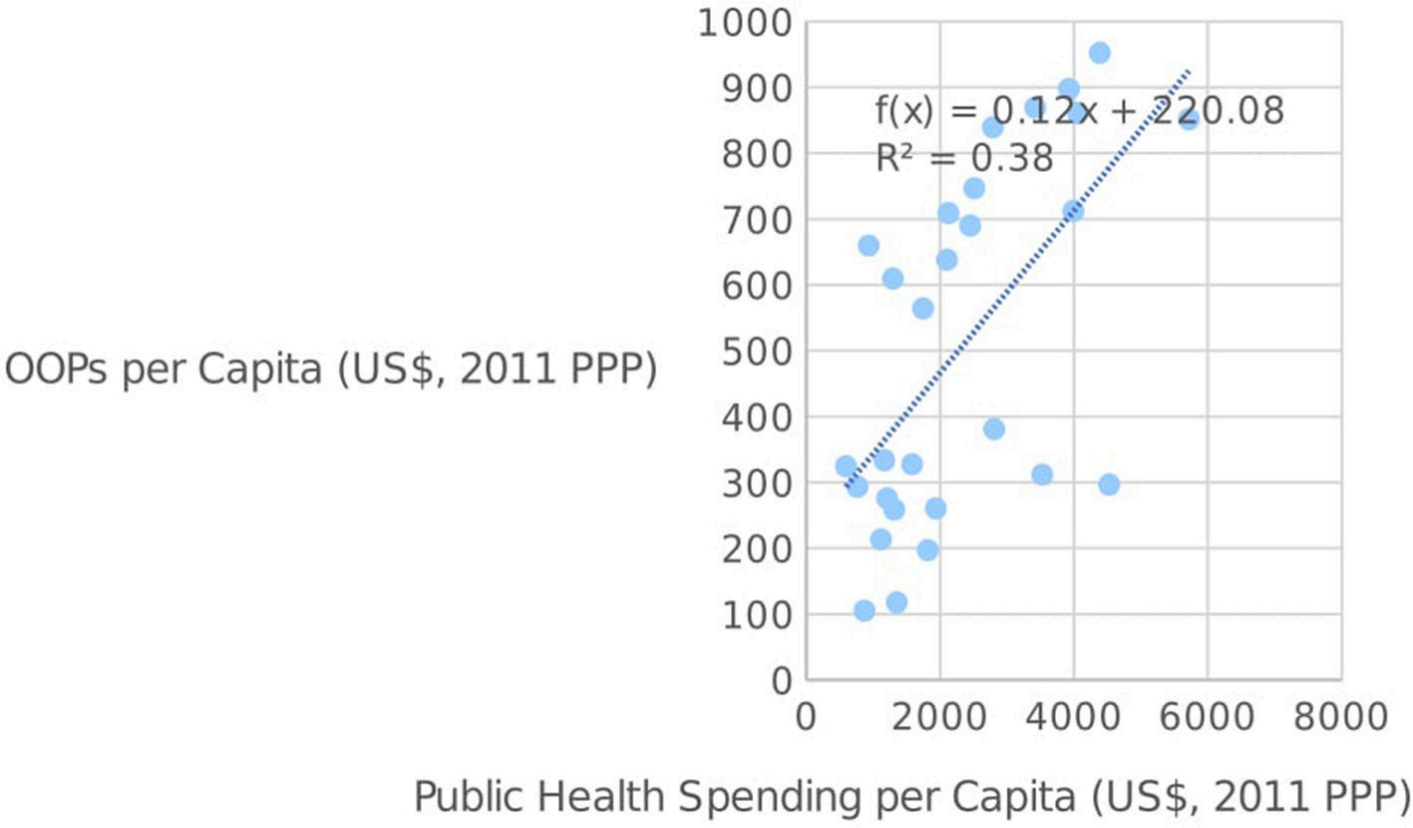
Public health spending per capita and OOPs per capita in the EU [source: (14)].

It is interesting to note the relationship between public health spending and OOPs among European countries. As seen in Figure 3 below, there seems to be a positive correlation between the two variables, thus indicating that OOPs are often complementary to public health spending, rather than a substitute, at least within the EU. The fact that this trend is reversed among our group of Mediterranean countries is particularly intriguing, since lower-than-average public health spending in these countries is being accompanied by higher-than-average OOPs, thus shifting the burden of healthcare provision from the state to the private individual.

We now turn to a more in-depth analysis of the economic indicators of the countries under consideration. In Malta, THE as a percentage of GDP increased from 5.7 to 9.7% between 1995 and 2014, the highest of any Mediterranean country. Furthermore, Malta also experienced the highest growth in THE per capita among the Mediterranean countries considered, with an increase of almost 242% from US$898.48 in 1995 to US$3071.63 in 2014 (Table 2). This is a significant development by the country when considering the country's size and population. Inter alia, OOP (Out of Pocket Expenditure) per capita has also increased from US$174 to US$709 over the designated time frame, while the share of private expenditure on health has remained broadly constant at 32–31%, albeit with some variation over the years (with some variants reaching 37% between 2008 and 2010). In fact, the share of public health expenditure has also remained broadly constant over this period, thereby indicating that Malta's growth in healthcare expenditure occurred across both private and public sectors.

**Table 2:** Select health expenditure indicators for mediterranean countries in 2014 [Source: (14)].

	**Total health expenditure per capita**	**Public spending in total expenditure**	**Private spending in total health expenditure**	**Proportion of total government expenditure on health**
Cyprus	US$2,062.37	45.23%	54.77%	6.90%
Greece	US$2,098.05	61.66%	38.34%	13.20%
Italy	US$3,238.89	75.61%	24.39%	14.70%
Malta	US$3,071.63	69.16%	30.84%	13.30%
Portugal	US$2,689.94	64.82%	35.18%	13.40%
Spain	US$2,965.82	70.88%	29.12%	15.40%

Portugal recorded a THE of 7.4% as a proportion of GDP in 1995, increasing to 9.5% by 2003 and staying relatively constant over the next decade. Per capita healthcare expenditure grew from US$1017.33 in 1995 to US$2689.94 in 2014, with minimal growth recorded since 2008. The public-private decomposition of healthcare has remained broadly constant over this period, with a minor decline in the private sector's share in healthcare expenditure from 37 to 35%, although it is important to note that the OOPS has increased from US$209 to US$563 within the period 1995–2014.

In Spain, THE as a proportion of GDP increased from 7 to 9% over the period under review, although this has dipped somewhat since 2010. The same can be said for per capita health expenditure, which peaked at US$3038.79 in 2009 before settling at US$2965.82 in 2014. The government's share of THE in Spain declined from 72% in 1995 to 70.9% in 2014, despite rising to a high of 75.7% in 2009, with the private sector mirroring these developments. This in part reflects the onset of the 2008-09 global economic crisis which had a significant impact in Spain both in terms of economic growth as well as government finances, with austerity measures implemented in order to rein in public spending and soaring debts.

Cyprus has the lowest level of healthcare expenditure in the Mediterranean, both as a proportion of GDP, as well as in per capita terms. Over the period under review, Cyprus recorded a THE of 4.7% of the GDP in 1995, which increased to 7.4% by 2014. Likewise, per capita expenditure rose from US$731.35 in 1995 to US$2062.37 in 2014, albeit following three consecutive years of decline. The government's share in THE has grown significantly over the period 1995 to 2014, from 35.8% to 45.2%, while the share of private expenditure has fallen from 64.2 to 54.7%. Nonetheless, Cyprus continues to be the country with the highest proportion of private health expenditure both in the Mediterranean and indeed the rest of the EU, and is the only country where the private sector accounts for the majority of healthcare spending.

Italy's THE reached 9.2% of GDP in 2014, up from 7.1% in 1995, although recent years have been characterized by very minimal growth and some minor declines, perhaps as a result of the global financial crisis. In fact, although Italy has the highest level of per capita healthcare expenditure in the Mediterranean (US$3238.89 in 2014, compared to US$1559.33 in 1995), this has been on a downward trajectory since 2011. The public sector's share in total healthcare expenditure has risen from 70.8% in 1995 to 75.6% in 2014, while the private sector's share has fallen from 29.2% in 1995 to 24.4% in 2014.

Greece's THE trajectory has largely mirrored the country's economic fortunes, particularly in the aftermath of the global financial crisis. In fact, as a percentage of GDP total healthcare expenditure actually fell slightly over the period 1995 to 2014, from 8.3 to 8.1%, with the decline mainly concentrated in the 2009-2014 period. Health expenditure per capita did increase over the period under review, from US$1266.563 in 1995 to US$2098.05 in 2014, the smallest increase in the Mediterranean, mainly as a result of a steep decline from US$3012.66 in 2008, coinciding with the economic crisis and subsequent austerity program. It is interesting to note the sectoral share of healthcare expenditure in Greece over this period, since in 1995 the private-public shares were relatively similar at 48 and 52% respectively. Since then however, the public sector's share increased to 61.7% in 2014, while the private sector's share has fallen to 38.3%.

## Discussion

The results presented above point toward a number of key similarities shared by the Mediterranean countries as regards healthcare expenditure. More specifically, we observed that THE, both in per capita terms and as a percentage of GDP, is lower in these countries relative to the EU average, mainly as a result of lower-than-average public health spending, albeit in most cases this has increased over time. OOP health expenditure is also higher in these countries relative to other European countries (18), underlining the importance of private initiatives in the provision of healthcare in the Mediterranean. This may be due to the culture of people within these countries to require ‘person-centered care’ which they might feel is not received through the services offered through public medical care (1). Although, the governments in these countries do provide free public health services, the waiting time required to receive these services and the personalized attention may not always meet the expectations of people requiring these services, who would thereby opt for private health care instead.

Furthermore, with the exception of Malta, THE across these countries has stalled somewhat in recent years (both in per capita terms and as a proportion of GDP), which can be linked to the 2008-09 global macro-economic and financial crisis and the impact that this had within these countries in terms of dampened economic conditions, as well as austerity measures to control public finances (18).

**Figure 4:**
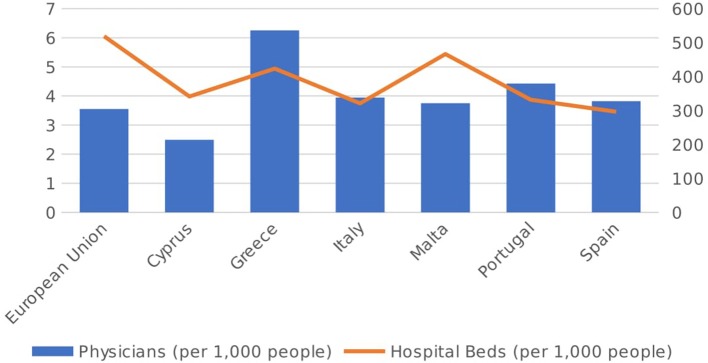
No. of physicians and hospital beds (per 1,000 people) in 2014 [Source: (14)].

**Figure 5:**
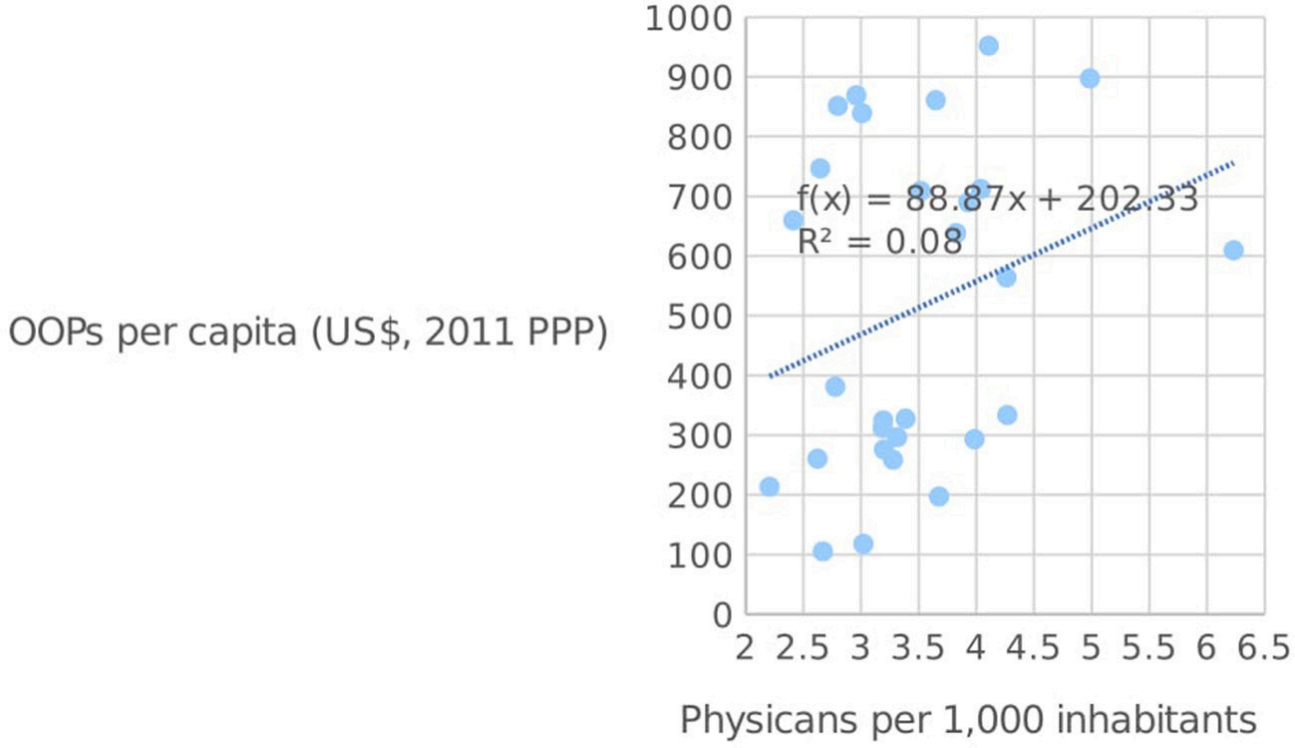
OOPs per capita and the number of physicians per 1,000 inhabitants in the EU [Source: (14)].

When analyzing whether these salient characteristics have had any discernible impact on the actual quality and infrastructure of healthcare in these six Mediterranean countries, we take note of (vide Figure 4) the number of physicians working in each country, together with the number of hospital beds (per 1,000 inhabitants), for the year 2014. It is clear that when looking at the supply of physicians, only Cyprus is lagging behind the EU average of 3.55 per 1,000 people, which may reflect the fact that it has the lowest level of healthcare expenditure in the Mediterranean. All other countries are above the EU average, with Greece leading the way with 6.3 physicians per 1,000 people. Thus it is clear that, for the vast majority of Mediterranean countries, the relatively high proportion of private sector financing coupled with lower-than-average healthcare spending does not translate into lower physician numbers, since most exceed the EU average. In fact, a simple scatter plot of the number of physicians per 1,000 inhabitants against OOPs per capita (Figure 5) shows that the two are generally positively-correlated within the EU, implying that countries with higher OOPs also have a higher proportion of physicians, perhaps reflecting the prevalence of private healthcare providers.

**Figure 6:**
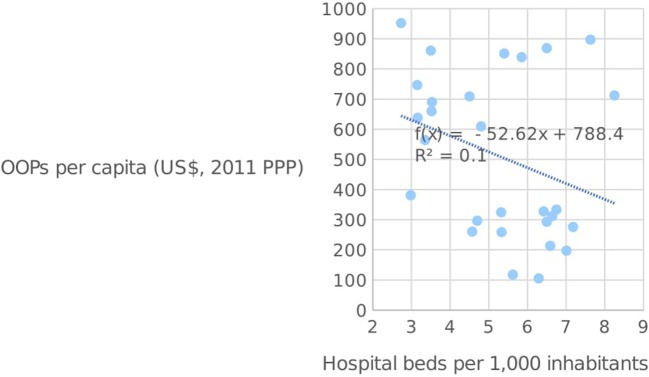
OOPs per capita and the number of hospital beds per 1,000 inhabitants in the EU [Source: (14)].

Matters are however somewhat different when it comes to the supply of hospital beds, since all six Mediterranean countries lag behind the EU average of 519.01 beds per 1,000 people. Malta has the highest number of hospital beds among this group, at 466.6 per 1,000 people, with Spain at the other end of the spectrum with 296.6. An adequate supply of hospital beds is a crucial component of any country's healthcare infrastructure (19), and may therefore have an impact on the quality of service provided. Figure 6 below shows the number of hospital beds plotted against OOPs per capita in the EU. As seen from the diagram, the two variables are negatively-correlated, which indicates that those countries with high OOPs tend to have a smaller supply of hospital beds. Although the direction of causation is somewhat open to debate, it may well be the case that high OOPs are simply the result of weak supply conditions as captured by the availability of hospital beds, which necessitate higher private expenditures in order to ensure quick service.

We then consider various cross-country indicators of healthcare quality to assess the Mediterranean countries' overall performance. We start with the World Health Organization's ranking of the world's health systems (20), which was issued in 2000 as a definitive guide to the quality of healthcare across its 191 member countries. All six Mediterranean countries ranked in the top 25 countries; indeed, Italy (2nd), Malta (5th), and Spain (7th) all ranked in the top ten, with only Cyprus somewhat lagging behind in 24th, which is still above several high income countries like Germany, Canada and the USA. Clearly, any tangible conclusions drawn from this ranking must be taken with some caution, both due to the controversial nature of this ranking system (21), which has never been updated, coupled with the dated nature of the ranking itself, which does not take into account more recent events such as the ramifications of the 2008-09 economic crisis (22). Nonetheless, at face value this ranking appears to show that the quality of healthcare systems within these six Mediterranean countries is in excellent shape, generally outperforming those in other European countries.

Another potential set of indicators is related to mortality rates and life expectancy at birth, which are both used in the computation of the United Nation's Human Development Index (23). Figure 6 plots the crude death rate in each country (per 1,000 inhabitants) and the life expectancy at birth. With regard to the death rate, things are somewhat mixed; three countries, namely Cyprus (6.8), Malta (7.7), and Spain (8.5) are all below the EU average of 9.73 deaths per 1,000 people, while the other three, namely Italy (9.8), Portugal (10.1), and Greece (10.4) are all above. Cyprus is of particular interest, given that it has both the lowest level of healthcare expenditure per capita and the lowest number of physicians per 1,000 inhabitants, and yet still has the lowest death rate, which may reflect other generally-favorable health conditions within the country. When it comes to life expectancy, all six Mediterranean countries are above the EU average of 80.9 years, with Spain leading the way at 83.3 years. Closely followed by Italy (83.2 years). Thus, these indicators do not highlight any significant issues when it comes to health outcomes in terms of deaths or life expectancy, although this ignores the incidence of illnesses and quality of life considerations.

The information observed above is adequate to draw a number of conclusions. It is backed by some secondary materials.

As with the rest of the countries under consideration, Malta has two healthcare systems (public and private), and practitioners are allowed to participate in the private system, as well as the public system. The public or government healthcare platform is free to all Maltese and European citizens (24). However, the private sector utilizes insurance policies to provide medical care to the patients who opt for it (25). Foreigners in Malta are encouraged to use the private healthcare system and are advised to take medical insurance for the same purpose (26). Financing in the healthcare sector is therefore predominately sourced from the public sector (24, 25). The quality of Malta's healthcare services is uniquely high and, as mentioned earlier, the country was ranked fifth by WHO in 2000 (20).

Portugal's healthcare system is driven by subsystems that merge the objectives of both the private and public healthcare systems (27). Some of these include mutual funds, private health insurance, and social health insurances models for specific professionals. The National Health Service (Serviço Nacional de Saúde, or SNS) is managed by the Ministry of Health which is also responsible for policy development (27). Changes in total healthcare expenditures in recent years are attributable to various developments, including government policies aimed at reducing public expenditures, the influx of immigrants to Portugal (28), as well as the 2005 primary healthcare reform which sought to improve the overall efficiency and effectiveness of the healthcare system by decentralizing the operation of State-run health centers through the establishment of autonomous Family Health Units (29).

In Spain, the publicly-funded Spanish Health Service (or SHS) provides free medical treatment for all patients, who undertake a consultation with a doctor who is recognized by or affiliated with the Spanish State Health Service (30), with coverage reaching 99.1% of the Spanish population in 2003 31. Otherwise, one is required to pay as a private patient or use private insurance to cover the bill (32). The healthcare system is largely funded by the government from social security and taxation receipts, although as seen earlier the private sector accounts for roughly a quarter of the overall industry (11, 32). Since 2002, the operation of the SHS has been completely devolved to the regional level, whereby each region is responsible both for the allocation of funds across different healthcare uses as well as the collection of revenues to fund the system. Despite these reforms, a number of key challenges remain, including improved integration and harmonization of healthcare across autonomous states, public accountability and financial sustainability in light of the economic crisis (33).

Cyprus has a dual healthcare system which, uniquely to the rest of the European countries, is largely skewed toward the private sector, although in recent years public health provision has grown in importance. Cyprus is the only EU member state without (near) universal public health coverage, with around 83% of the population in 2014 covered by free healthcare (12). The nature of the current healthcare system in Cyprus encourages residents to take up private insurance since the public system is largely inefficient, with problems related to coordination, waste of resources and corruption in public hospitals (24, 34). In 2013 the Cypriot government, upon the recommendation of the European Central Bank, the International Monetary Fund and the European Commission, announced its intention to reform the health service and establish a National Health Insurance Scheme (NHIS), with the aim of ensuring universal coverage (35). The new scheme was approved by the Cypriot Parliament in June 2017, and full implementation is expected by 2020. Under the new system, patients will be allowed a choice across both private and public health service providers, and everyone will have access to all medical interventions regardless of actual contribution levels, with funding coming from government taxes, worker contributions and employers.

Healthcare in Italy is largely operated by the public sector, and has the second highest-ranked health service in the world according to the World Health Organization (20). The national health service, or the Sistema Sanitario Nazionale (SSN), has been in operation since 1978 and provides universal healthcare to all citizens. Similar to Spain, administrative, delivery and financing responsibilities have largely been devolved to each of the country's 20 regions, a process which has been ongoing since the early 1990s. Despite the impressive results obtained, the SSN is currently facing a number of important challenges, notably as a result of the economic crisis and associated pressures on public finances 36. This has resulted in various cost-cutting initiatives within the healthcare sector, such as a reduction in health funding of €4.7 billion between 2012 and 2014, lower expenditure caps on health equipment in 2010 as well as the reintroduction of a €10 charge for all outpatients (37). Improved harmonization and coordination across the disparate regional healthcare providers also remains a priority, due to significant heterogeneity in the quality of health services across autonomous regions (38).

In Greece, public healthcare is provided by the National Healthcare Service (ESY), built on the principle of universal coverage (39). A public insurance company IKA (Idrima Kinonikon Asfaliseon) is entrusted with administering the social security system in Greece. Free medical services are provided on the basis of a patient providing relevant applicable certification to an IKA office where health services books are issued and referrals to medical facilities housed under IKA are made. One might be required to pay fees for secondary assessments such as x-rays and certain medications which are not entirely free (39). Therefore, the IKA scheme serves to assist the residents in taking care of hospital expenses and not fully rendering free services. Another issue of concern was the extensive growth of Greek Pharmaceutical market largely driven by unsustainable policies in dispensing and reimbursement of medicines (40).

Despite the high number of physicians per 1,000 people, the Greek public health system has long been plagued by inefficiencies in management and organization, requiring significant reforms (41). The global economic crisis has undoubtedly had the largest impact on Greece's public healthcare system in recent years (42, 43). In the wake of the crisis, total spending on healthcare (in real terms) shrank by over a third between 2009 and 2013, mainly as a result of government funding cuts due to austerity measures aimed at controlling spiraling public debt levels, including a €1.8 billion cut in pharmaceutical expenditure between 2009 and 2013 (12). Furthermore, reforms enacted in 2011 saw the creation of the new National Health Services Organization (EOPYY) instead of IKA, which sought to move away from the idea of universal coverage to entitlement on the basis of insurance status. With unemployment rates reaching unprecedented levels (over 27% in 2013), this lead to several people losing their entitlement status, while many self-employed ceased to renew their plans amidst worsening economic conditions (44). The Greek government has sought to tackle this problem directly by enacting a new policy in 2014, extending coverage to the uninsured for prescription drugs, emergency services in government hospitals and certain non-emergency care, with further extensions to coverage enacted in 2016 for certain vulnerable groups like the registered unemployed and refugees.

## Study limitations

The study incorporated information from different sources to ensure that it maintained accurate information for the analysis required. However, the time period considered, 1995-2014, is an accurate long-run indicator. Additionally, one must also take into account the unique factors that characterized both the last quarter of the twentith century, including mass development of industries, cities and economies (45), as well as the onset of the global economic crisis in 2008-09, which may limit the generalizability of these findings to other territories or time periods.

It is also arguable that the reports filed by individual countries through their various agencies might be fixed and, therefore, unreliable (46). We sought to mitigate against such occurrences through the use of data provided by international organizations like the World Health Organization to ensure objectivity and comparability throughout this study.

## Conclusion

With this study, we have sought to analyze healthcare provision and expenditure across six Mediterranean countries (including Portugal) forming part of the European Union. To this end, we considered various indicators and statistics in order to derive commonalities and differences across these countries. We compared their overall trends to those observed across the rest of the European Union. We then analyzed these findings in light of other data related to the quality of healthcare and infrastructure within these countries, before looking at recent developments within each country and the main challenges faced.

The results show that on average, the Mediterranean countries spend less on total healthcare expenditure than the EU average, both as a proportion of GDP as well as in per capita terms. This is primarily driven by lower-than-average public funding of healthcare, which must be considered in light of the economic crisis which had a significant impact on the countries under review, and reductions in public spending due to austerity measures. On the flipside, the Mediterranean countries have a higher presence of private health providers in total funding, while OOP health expenditures are higher in these countries relative to the average in the EU.

With regard to the overall health infrastructure in these countries, we observed that although the supply of physicians is largely in line with the rest of the EU, there is under-supply when it comes to hospital beds, which may be symptomatic of lower government funding and thus driving higher OOPs. Nonetheless, all countries score highly when it comes to evaluating the quality of the health services, both according to independent international rankings like the WHO's 2000 metric as well as other indicators like mortality rates and life expectancy, which all point toward favorable health outcomes in the Mediterranean.

Many of the countries analyzed in this paper have undertaken significant healthcare reforms in recent years, with the aim of improving efficiency and quality in healthcare provision and thereby decreasing the high out-of-pocket healthcare spending. The economic crisis has stifled these reforms to some extent, since in many countries like Greece and Italy significant cuts to public health expenditure were enacted in order to rein in rising debt levels. Therefore, a major challenge faced by these countries is to ensure maximum coverage for citizens, particularly the most vulnerable members of society, all while faced with tightening public health budgets. This further emphasizes the importance of public sector reforms in terms of ensuring improved efficiency, governance and management of public health administration. Furthermore, increased decentralization and autonomy of public health management at the regional level, which is a feature of many countries in the Mediterranean and indeed across Europe, brings new challenges like ensuring coordination across regions and consistency of quality standards to avoid the creation of regional disparities in healthcare provision, which in turn may exacerbate existing disparities in economic and social outcomes.

**Figure 7:**
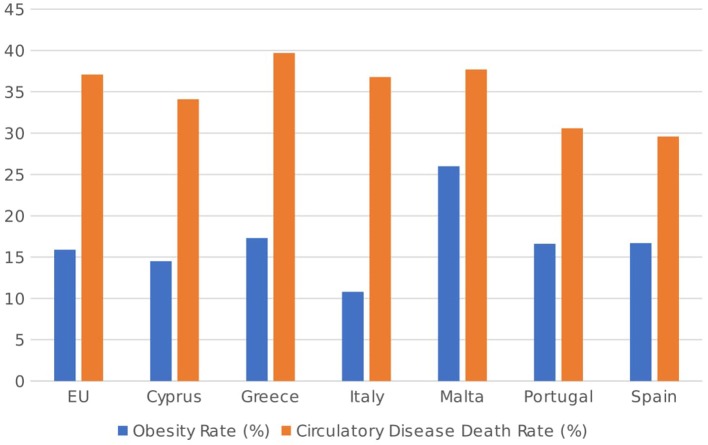
Obesity rates (as % of population aged 18 or over) and circulatory disease death rates (as % of total deaths) in 2014 [Source: (49, 50)].

Besides these financial and administrative challenges, various socio-economic and cultural developments across the Mediterranean may have important implications for healthcare systems in these countries. One such development is increased migratory flows from North African countries, which have grown significantly over the last two decades and which place further pressures on public healthcare and the need for universal coverage (47). Another factor is the growing proportion of over-60 citizens in the population of most developed countries, including those in the Mediterranean (48), which will necessitate increased investment in specialized care and infrastructure to cater for these specific needs. Finally, the increased incidence of cardiovascular diseases and diabetes, often associated with lifestyle factors and obesity, must also be tackled by the relevant health authorities. Despite the apparent health benefits of the much-lauded Mediterranean diet, the obesity rate in Greece (17.3%), Malta (26%—the highest in the EU), Portugal (16.6%) and Spain (16.7%), as shown in Figure 7, is above the EU average of 15.9% of the adult population, while the number of deaths caused by circulatory diseases is also higher than the EU average of 37.1% of deaths in Greece (39.7%) and Malta (37.7%). Thus, public health interventions aimed at both preventing and treating the onset of obesity and circulatory diseases is a leading priority for most of these countries.

These findings further serve to emphasize the commonalities observed in this group of EU Mediterranean countries, who despite their diversity have clear similarities in terms of socio-demographic developments, health outcomes, total health expenditure patterns as well as the relative importance of private healthcare providers in these countries. It is the intention of the authors to invite future research, most likely using the health care utilization model or related health sociology models to explain the likelihood for people in these countries to go for OOP expenditure.

## Author contributions

All authors listed have made a substantial, direct and intellectual contribution to the work, and approved it for publication.

### Conflict of interest statement

The authors declare that the research was conducted in the absence of any commercial or financial relationships that could be construed as a potential conflict of interest. The reviewer KK and the handling Editor declared their shared affiliation.
